# Supplementary material for: The effects of different rootstocks on aroma components, activities and genes expression of aroma-related enzymes in oriental melon fruit
Source: PeerJ. 2024 Jan 5;12:e16704. doi: 10.7717/peerj.16704 (PMC10773451; doi:10.7717/peerj.16704)
Supplement: Supplemental Information 1 [file peerj-12-16704-s001.docx]

MIQE checklist for authors, reviewers, and editors

**Experimental design**

Definition of experimental and control groups

The oriental melon variety ‘QingxianYangjiaocui’ was used as a scion, while ‘Qinmi No.1’ (*Cucumis melo* L.) and ‘Ribenxuesong’ (*Cucurbita maxima* Duch.) were used as rootstocks. The fruits of the self-grafted plants of ‘QingxianYangjiaocui’ were used as the control group, while the fruits of the grafted plants of ‘Qinmi No.1’ and ‘Ribenxuesong’ rootstocks were used as the experimental group.

Number within each group

At the end of the fruit expansion period (30 days after pollination, 30DAP) and the commercial ripening period (42 days after pollination, 42DAP), five plants were randomly selected from each plot, and 3 fruits of each plant were taken and crosscut.

**Sample**

Description

Grafting was carried out on full opening of the first true leaves of the rootstocks and initial exposure of the first true leaves of the scion. The grafted seedlings were transplanted to the greenhouse 1month post-grafting and arranged in randomized groups. Each experimental groups comprised three biological replicates of 20 plants, which were raised using standard management practices. Hand-pollinated bisexual flowers were marked, with three of the subsequently developing fruits being retained on each plant. At the end of the fruit expansion period and the commercial ripening period, five plants were randomly selected from each plot, and 3 fruits of each plant were taken and crosscut.

Microdissection or macrodissection

All samples were collected through macrodissection.

Processing procedure

The central portions (2/5) of each fruit were extracted for determination of the genes expression level of alcohol dehydrogenase (ADH) and alcohol acyltransferase (AAT).

If frozen, how and how quickly?

Liquid nitrogen used for freezing fruit samples.

If fixed, with what and how quickly?

Liquid nitrogen used for fruit sample fixation.

Sample storage conditions and duration

The sample is stored in a -80 ℃ refrigerator.

**Nucleic acid extraction**

Name of kit and details of any modifications

Huayueyang Quick RNA Isolation Kit

Details of DNase or RNase treatment

During the process of extracting RNA, wear gloves, experimental clothing, and RNase free with gun tip and centrifuge tube, respectively. Add 300 μL to the centrifuge tube Deproteinization solution of and 200 μL prepared chloroform, shaken on an oscillator for 30 seconds, mixed well, centrifuged at room temperature of 12000 g for 10 minutes. The supernatant was transferred to another clean 1.5 mL plastic centrifuge tube, and an equal volume of rinsing solution was added. The mixture was thoroughly inverted and mixed well. Add the obtained mixture into the same centrifugal adsorption column twice (each time with a volume not exceeding 700 uL), and centrifuge 12000 g at room temperature for 1 minute after adding the adsorption column. Discard the penetrating solution. Add 500 μL washing solution to the adsorption column washing solution, repeat. Then centrifuge at room temperature of 12000 g for 1 minute to remove residual liquid. Take 50 μL of membrane digested DNA and add it to an adsorption column, and let it sit at room temperature for 30 minutes. Add 500 μL of enzyme removal solution to the adsorption column, mix well, and centrifuge at 12000 rpm for 1 minute at room temperature. Repeat the above steps after pouring the waste liquid, and then centrifuge at 12000 rpm for 2 minutes at room temperature.

Instrument and method

Calculate RNA content by measuring the absorbance value of RNA solution at 260nm using Thermo Scientific NanoDrop 2000 | 2000c Spectrophotometers, with a sample size of 1 μL each time.

**Reverse transcription**

Complete reaction conditions

FastGng RT Kit (With gDNase) was used for RNA reverse transcription.

Amount of RNA and reaction volume

The reaction liquid includes 2 μL 10 × Fast RT Buffer (SYBR Green), 2 μL RT Enzyme Mix, 2 μL FQ-RT Primer Mix, and finally add RNase Free ddH_2_O to 10 μL.

Priming oligonucleotide (if using GSP) and concentration

| **Name** | \| **Oligonucleotide sequence** \| \| \| --- \| --- \| \| **Forward primer sequence (5’-3’)** \| **Reverse primer sequence (5’-3’)** \| | |
| --- | --- | --- | --- | --- | --- | --- |
| *β-actin* | CCGTTCTGTCCCTCTATGCT | AGTAAGGTCACGACCAGCAA |

Primer concentration is 10 μM.

Temperature and time

Set the reverse transcription temperature to 50 ℃, reaction time to 15 minutes, cycle once, 95 ℃ reaction for 3 minutes, cycle once.

**qPCR oligonucleotides**

Primer sequences

| **Name** | \| **Oligonucleotide sequence** \| \| \| --- \| --- \| \| **Forward primer sequence (5’-3’)** \| **Reverse primer sequence (5’-3’)** \| | |
| --- | --- | --- | --- | --- | --- | --- |
| *CmAAT1* | CCACAGGGGCCAGAATTAC | TGGAGGAGGCAAGCATAGACT |
| *CmAAT2* | CTATAATTGGAGGGTGTGGAATTATC | AACATTTGCCCTAAATCTTTCCAT |
| *CmADH1* | GTGTTCTTAGCTGCGGCATTT | TTGACCCTTTTTAGGCTTTGCA |
| *CmADH2* | GCGGAATCGTTAAAGGGTGTA | AGCCGCCTCTCTCTCTTCTTC |
| *β-actin* | CCGTTCTGTCCCTCTATGCT | AGTAAGGTCACGACCAGCAA |

**qPCR protocol**

Reaction volume and amount of cDNA/DNA

Configure a 20 μL reaction system, mainly including 10 μL2 × M5 HiPer SYBR Premix EsTaq (withTli RNaseH), 0.8 μL primer,7.6 μL ddH_2_O and 1.6 μL template DNA.

Buffer/kit identity and manufacturer

2×M5 HiPer SYBR Premix EsTaq (with Til RnaseH)

Complete thermocycling parameters

| Program | Cycles | Steps |
| --- | --- | --- |
| Preincubation | 1 | 95℃ for 60s |
| 3 step Amplification | 40 | 95℃ for 5s |
|  |  | 60℃ for 10s |
|  |  | 72℃ for 15s |
| Melting | 1 | 95℃ for 10s |
|  |  | 65℃ for 60s |
|  |  | 97℃ for 1s |

Manufacturer of qPCR instrument

Roche lightcycle 96 real-time PCR system (Applied Biosystems, USA)

**Data analysis**

qPCR analysis program (source, version)

LightCycler® 96 SW 1.1

Method of C_q_ determination

The 2^-△△Ct^ method is used for the C_q_ determination.

Justification of number and choice of reference genes

The *β-actin* was used as an internal reference control gene to standardize gene expression data.

Number and stage (reverse transcription or qPCR) of technical replicates

qPCR has 3 technical replicates.

Repeatability (intraassay variation)

Intraassay variation ≤ 1.

Statistical methods for results significance

For gene expression data, significant differences were determined based on a one-way ANOVA and Duncan's multiple range test at the *p* < 0.05 level.

Software (source, version)

The data were analyzed with an analysis of variance (ANOVA) using the SPSS 25.0 statistical package.
